# Response to “Antiviral RNA silencing in mammals: the importance of selecting the appropriate experimental model”

**DOI:** 10.1093/nar/gkaf736

**Published:** 2025-08-11

**Authors:** Petr Svoboda, Radek Malik

**Affiliations:** Institute of Molecular Genetics of the Czech Academy of Sciences, Videnska 1083, 142 20 Prague 4, Czech Republic; Institute of Molecular Genetics of the Czech Academy of Sciences, Videnska 1083, 142 20 Prague 4, Czech Republic

## Abstract

In their commentary *Antiviral RNA silencing in mammals: the importance of selecting the appropriate experimental model*, Xing Liu and Yang Li critically review our recent work by Kulmann *et al.* published in *Nucleic Acids Research*. While we agree with authors on a number of their points, especially those concerning complexity of virus–host systems, we would like to add some missing information and clarify several important aspects of our animal model system and mammalian canonical RNA interference in general.

## Introduction

Canonical RNA interference (RNAi), one of small RNA pathways, is defined as sequence-specific mRNA degradation guided by small interfering RNAs (siRNAs) made by RNase III Dicer from long double-stranded RNA (dsRNA) [1]. RNAi roles in animals include gene regulation, antiviral immunity, or defense against transposable elements. However, multiple independent lines of evidence show that RNAi became a rudimentary pathway in the lineage leading to mammals as a different dsRNA-induced innate immunity evolved (reviewed for example in [2]), while mammalian Dicer acquired adaptations for producing gene-regulatory microRNA and lost processivity needed for efficient siRNA production [3–7]. The only known example of functionally essential endogenous mammalian RNAi evolved in mouse oocytes, which express a specific truncated Dicer variant needed for RNAi-mediated suppression of retrotransposons and gene expression [8–11]. Importantly, RNAi is not entirely absent in other mammalian cells and was repeatedly detected, particularly under specific conditions, which overcome common obstructions in somatic cells such as sequence-independent dsRNA responses, inefficient siRNA production by full-length Dicer, and additional miRNA-pathway related hindrances. It is thus not surprising that some authors observed antiviral effects of mammalian RNAi, while others did not.

Our approach to the issue of mammalian canonical RNAi activity stemmed from what we have learnt from activated RNAi in mouse oocytes. The pinnacle of our effort was production and validation of *Dicer*^ΔHEL1^ mouse model [6], where the endogenous *Dicer* locus was engineered to produce the truncated Dicer variant supporting RNAi in mouse oocytes. Because homozygous mouse mutants are not viable (one allele encoding full-length Dicer seems to be required for fidelity of miRNA biogenesis), heterozygotes must be used for *in vivo* analyses. Thus, what our lab contributed to understanding mammalian RNAi was successful development of a mouse model with genetically enhanced RNAi in order to address two questions: (i) How well RNAi works *in vivo* if we boost siRNA production from dsRNA by an order of magnitude? (ii) Will this have any antiviral effect? We addressed both questions in two articles. The first one was addressed in Buccheri *et al. Functional canonical RNAi in mice expressing a truncated Dicer isoform and long dsRNA* [12]. To test the second one, we infected *Dicer*^ΔHEL1/wt^ mice with four different viruses CVB3, TBEV, EMCV, and LCMV and, as described in Kulmann et al*. Enhanced RNAi does not provide efficient innate antiviral immunity in mice* [13], we did not observe any remarkable antiviral effect.

We would like to bring up four specific points about our work on mammalian RNAi:

- Xing Liu and Yang Li state “Theoretically, *Dicer^ΔHEL1/wt^* mice should exhibit an enhanced RNAi response, leading to the production of higher levels of Dicer-processed siRNAs.” This statement is misleading and incorrect. *Dicer^ΔHEL1/wt^* mice do exhibit enhanced RNAi response. We have systematically analyzed siRNA production and canonical RNAi in these mice and dedicated the above-mentioned work by Buccheri *et al.* [12] to it. We showed that (a) *Dicer^ΔHEL1/wt^* mice exhibit markedly increased siRNA production in all tested organs, (b) siRNA production depends on dsRNA availability, (c) only very high siRNA levels (at par with abundant miRNAs) were able to induce detectable target repression *in vivo*, and (d) even endogenous full-length Dicer might be able to mediate RNAi *in vivo* with high dsRNA availability. Buccheri *et al.* paper is critical for understanding *in vivo* limits of the mammalian canonical RNAi and should not be omitted from discussion on RNAi in *Dicer^ΔHEL1/wt^* mice.
- We did not aim for selecting the right virus or the appropriate viral infection model and we see it as a positive aspect of our work. We simply examined four different viral infection models available in collaborating laboratories, which were willing to check the antiviral resistance of *Dicer^ΔHEL1/wt^* mice using their routine infection protocols. It is possible that when using other viruses, a different genetic background of mice or with some fine-tuned infection protocols even with viruses we used, an antiviral effect would be observed. This would not be mutually exclusive with our observations and conclusions.
- Our unique and well-characterized *Dicer^ΔHEL1/wt^* mice (or frozen sperm) are freely available to anyone for further antiviral RNAi research. The model is as good as enhanced RNAi *in vivo* in mice can be precisely engineered endogenous Dicer allele to remove the HEL1 domain while retaining endogenous control of Dicer expression (off note: as a control, we also produced a full-length Dicer variant called SOM, which is lacking just introns removed in the *Δ*HEL1 mutant). Furthermore, *Dicer^ΔHEL1/wt^* mice can be combined with other mutants, such as those of innate immunity dsRNA sensors; we have only examined PKR loss [12, 13]. Notably, the above-mentioned SOM Dicer variant produced as a control for the loss of N-terminal introns [6, 12], would also lack the enigmatic AviD Dicer proposed to protect tissue stem cells against viruses [14] and thus would be an excellent genetic model to test the hypothesis that AviD Dicer is physiologically relevant.
- Any negative bioinformatics siRNA analysis of total small RNA-seq data should be interpreted with caution. For example, analysis of phasing and duplexes is useful for detecting phased siRNA production from (viral) dsRNA termini while absence of phasing and duplexes does not prove absence of siRNAs since Dicer is able to cleave dsRNA internally too—this internal cleavage has been observed *in vitro* (e.g. [4]) and likely initiates siRNA production in cells and mice in our experiments with expressed long dsRNA hairpins. These long dsRNA hairpins are not accessible by Dicer from termini of the dsRNA stem while they induce RNAi and are reproducibly decorated along the dsRNA region with 21–23 nt siRNAs [10, 12, 15]. Similarly, absence of 21–23 nt nucleotide peak above the background of degradation products or lack of enrichment of terminal nucleotides in total small RNA-seq may not be sufficient evidence of absence of siRNAs. We think the best strategy for these situations is experimental analysis of AGO-bound small RNAs.

Taken together, we think that the core of the mammalian canonical (antiviral) RNAi problem is that suitable conditions for mammalian canonical RNAi rarely exist naturally and even when manipulating the system, it is challenging to induce efficient canonical RNAi in cultured cells and in mice. This notion is not mutually exclusive with works where authors managed to establish conditions to observe mammalian canonical (antiviral) RNAi. And we wish someone would be brave enough to use our mouse models to examine their viral models and report what they would find.
